# Surface-enhanced Raman spectra of medicines with large-scale self-assembled silver nanoparticle films based on the modified coffee ring effect

**DOI:** 10.1186/1556-276X-9-87

**Published:** 2014-02-19

**Authors:** Weiping Zhou, Anming Hu, Shi Bai, Ying Ma, Quanshuang Su

**Affiliations:** 1 Institute of Laser Engineering, Beijing University of Technology, Beijing 100124, China; 2 Department of Mechanical, Aerospace, and Biomedical Engineering, University of Tennessee, 509 Doughty Engineering Building, 1512 Middle Drive, Knoxville, TN 37996, USA

**Keywords:** Silver nanoparticle film, Coffee ring effect, SERS

## Abstract

We report here a simple and innovative method to prepare large-scale silver nanoparticle films based on the controlled coffee ring effect. It is demonstrated that the films can be used as surface-enhanced Raman scattering probes to detect low-concentration medicines. Silver nanoparticles with the average size about 70 nm were prepared by reduction of silver nitride. In our experiment, the coffee ring effect was controlled by tilting the substrates during the deposition of silver nanoparticle films. Silver nanoparticle films were spontaneously formed on the surface of silicon substrates at the temperatures about 50°C based on the solvent evaporation and the coffee ring effect. The microstructure of the films was investigated using the scanning electron microscope and atomic force microscope. The surface roughness of the films is found as small as 20 nm. Then, the films were exposed to aqueous solutions of medicine at different concentrations. A comparison with a Raman spectra measured with a conventional Raman spectrometer showed that the Raman signal can be detected in the solution with concentrations as low as 1 × 10^−5^ M, and the enhancement factor achieved by the silver nanoparticle film can at least reach to 1.08 × 10^4^. Our experimental results indicate that this technique is promising in the production of large-scale silver nanoparticle films for the surface-enhanced Raman scattering. These may be utilized in biochemical and trace analytical applications.

## Background

Raman spectroscopy is an important analytical technique for chemical and biological analysis due to the wealth of information on molecular structures, surface processes, and interface reactions that can be extracted from Raman spectra [1]. The Raman cross section of a normal Raman spectroscopy is inherently weak, thus preventing from the application of high-sensitivity analysis. Fortunately, for the last three decades, Raman techniques have experienced increasing application in many fields due to the observations of the enormous Raman enhancement of molecules adsorbed on special metallic surfaces. In 1974, it was first reported that an unusually strong enhanced Raman scattering signal occurred with pyridine molecules adsorbed on silver electrode surfaces that had been roughened electrochemically by oxidation-reduction cycles [2]. It was discovered that this process may enhance Raman activities at a 10^6^-fold at an appropriately prepared coinage metal surface.

Since its discovery in 1970s, surface-enhanced Raman spectroscopy (SERS) is becoming more attractive for applications, and it is fast moving from fundamental research to analytical applications in the biomedical and environmental areas [3]. The further development of SERS is mainly limited by the reproducible preparation of clean and highly active substrates [4]. The original substrates for SERS were electrochemically roughened metal electrodes [2]. Metallic nanoparticle films were used shortly after the discovery of the SERS effect and became the most studied class of substrates. Up to date, the SERS probes can be arbitrarily classified in three categories: (1) metallic nanoparticles in suspension, (2) metallic nanoparticles immobilized on solid substrates, and (3) nanostructures fabricated directly on solid substrates, which include nanolithography, template synthesis of nanostructures, pulsed laser deposition, and laser lithography [5-8].

The application of dispersed and aggregated metallic nanoparticles as a SERS probe in a real analytical problem is limited due to the poor reproducibility. The reproducibility problem can be mitigated by immobilizing the metallic nanoparticles on some kind of solid support [9]. Since the report of a SERS substrate consisting of metallic nanoparticles synthesized by a wet chemistry method and subsequently immobilized onto a solid support [10], the procedure gained popularity. Several works have been published based on this approach and its variations [8,11-13]. The attempts of using self-assembled metallic nanoparticles on solid support as a SERS substrate was reported by the Natan group in 1995 [9]. The self-assembly of metallic nanoparticles onto solid surfaces based on electrostatic attraction using polymers [14-16] and biomolecules [17,18] has also been widely reported, such as poly(vinylpyridine) which was used to immobilize Ag nanoparticles onto continuous Ag films [19]. Bifunctional SERS-active single microsize particles can be fabricated through the electrostatic-induced self-assembly. For example, Spuch-Calvar et al. [20] reported the fabrication of SERS and magnetic bifunctional spindle particles using polyelectrolyte as the linking reagent. Although the chemical and electrostatic self-assemblies are popular for fabricating SERS substrates, different approaches have also been explored. For example, capillary forces, dominant during the evaporation of a liquid droplet, can be used to drive the assembly of metallic nanoparticles [21-23]. The Halas group [20] used a drop-dry method to assemble a film of CTAB-capped nanoparticles on silicon wafers.

We report here a simple method to prepare large-area silver (Ag) nanoparticle films based on the coffee ring effect for the use of SERS. The ‘coffee ring effect’ is widely known as a typical evaporation-driven self-assembly and self-organization [24]. When a droplet of solutions containing nonvolatile solutes (e.g., coffee particles) dries on a substrate, it leaves a dense, ring-like deposit of the solutes, i.e., a ‘coffee ring,’ along the perimeter. In an industrial inkjet printing [25,26] and a biological application [27], a uniform pattern is usually required. The ‘coffee stain effect’ is an undesirable phenomenon. Thus, some efforts were spent to eliminate the coffee ring effect by changing the shape of the suspended particles [28]. In this paper, we show an innovative method to control the coffee ring effect by simply tilting the substrate and thereby obtaining a large-scale silver nanoparticle film. Moreover, the film can be applied as substrates for SERS to detect medicines. 5-Fluorouracil was selected as a model drug in this experiment since 5-fluorouracil-containing solutions and creams are extensively used in human patients for the treatment of solar and actinic keratoses and some superficial skin tumors. 5-Fluorouracil, an antimetabolite, is also used in veterinary medicine for the treatment of some cancers [29,30]. Drug content in the solution of a low concentration can be detected according to our experimental results. Our experimental results indicate that this self-assembly method shows great promise in the production of large-scale metallic films. These may be utilized in biochemical sensing and optical processing applications.

## Methods

### Preparation of silver nanoparticles

Silver nitrate (AgNO_3_), sodium citrate dehydrate, and deionized water, all in analytical grade, were used without further purification. Ag nanoparticles used in this study were prepared by an aqueous method as follows: Ag nanoparticles were synthesized through a chemical reduction reaction of AgNO_3_ using sodium citrate dehydrate as reducing agent. Details of the synthesis procedure have been presented in a previous study [31]. A solution of AgNO_3_ (1 mM) in 250-mL ultrapure water was heated to 80°C. A volume of 10-mL aqueous solution of Na_3_C_6_H_5_O_7_ · 2H_2_O (0.34 mM) was then added to the AgNO_3_ solution. Heating was continued to 90°C for 30 min after adding the citrate solution. The color of the solution changed from the colorless water to yellow after 15 min of heating and to gray after 25 min. The resulting sol is simply silver nanoparticles coated with organic shell, dispersed in water at a concentration of 1 mM [32,33].

### Preparation of silver nanoparticle solution with different concentrations

The different concentrations of the silver nanoparticle solution were fabricated by increasing the concentration of the silver nanoparticle solution from 1 mM to 0.1 M by centrifugation. Centrifugation was conducted at 9,000 revolutions per minute (rpm) for 5 min in 10-mL centrifuge tubes. The water was extracted from the centrifuge tubes using a pipette, leaving aqueous-based Ag nanoparticle paste at the bottom. Shock the tube to make the nanoparticle paste back into suspension, then collect the rest of the solution for the next centrifugation. Repeat this process until the required concentration solution was obtained.

### Preparation of silver nanoparticle films on silica substrates

**Figure 1:**
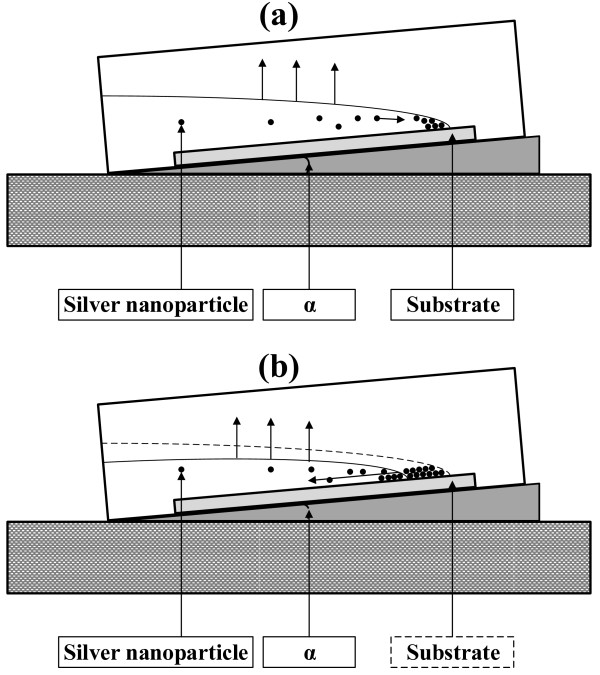
Schematic illustration of silver nanoparticles self-assembled on silica substrate (a, b).

Silicon wafers with single side polished were cut into required size, depending on the demand. The prepared silicon wafers were cleaned by an ultrasonic cleaning machine using deionized water for 10 min. These silicon wafers were then laid in a container, and the container was placed on an inclined platform with the angle of inclination *α* = 10°. The schematic of this device is shown in Figure 1. The solution of silver nanoparticles prepared with different concentrations was poured into the container. The evaporation was carried out inside an oven. This oven temperature was set to 50°C. After evaporation of the solvent, the self-assembled silver nanoparticle film was obtained.

### Characterization techniques

The absorption spectrum of the silver colloid was obtained using a UV-vis (UV-9000S, Shanghai Metash Instruments Co., Ltd., China) spectrophotometer. The morphology of the silver nanoparticles was examined by transmission electron microscopy (TEM; JEM-2010, JEOL Ltd., Akishima, Tokyo, Japan). The silver nanoparticle films were imaged using a scanning electron microscope (SEM; XL30 S-FEG, FEI Co., Hillsboro, OR, USA). The cross-sectional profiles of the silver nanoparticle films were measured using an atomic force microscope (AFM; Pico Scan TM 2500, Scientec, Les Ulis, France) and a Veeco surface profiler (Wyko NT1100, Veeco Instruments Inc., Plainview, NY, USA).

### Applications for surface-enhanced Raman spectroscopy

The silver nanoparticle film on silicon wafers was used as a substrate for surface-enhanced Raman spectroscopy. 5-Fluorouracil was dissolved in water using an ultrasonic cleaning machine for 5 min. 5-Fluorouracil is sparingly soluble in water [34]. In our experiment, the concentration of solution 1 × 10^−1^ M was not obtained because of the low solubility of 5-fluorouracil at room temperature. The concentrations of the solution were prepared as 1 × 10^−2^ M, 1 × 10^−3^ M, and down to 1 × 10^−6^ M. Then, the solution was dropped on the substrate for Raman detection. The SERS signal was measured with a commercial Raman equipment (inVia-Reflex, Renishaw, Gloucestershire, UK) using a laser with a 532-nm wavelength as the excitation source; the measuring laser spot size was about 3 μm, and the acquisition time was 10 s.

## Results and discussion

**Figure 2:**
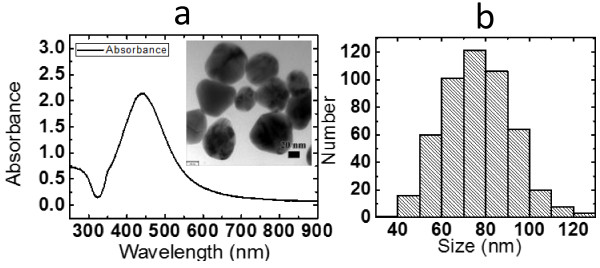
**Absorption spectra and particle size distribution of nanoparticles. (a)** Absorption spectra of silver nanoparticles. The inset shows the image of silver nanoparticles obtained by transmission electron microscopy; the scale of the image is 20 nm. **(b)** The particle size distribution of 500 nanoparticles.

Figure 2a shows the UV-vis absorption spectrum and a typical TEM image of silver nanoparticle suspension. It can be seen from the figure that the strongest peak appears at 440 nm, and a shoulder appears at 360 nm. The absorption spectra for the 40-nm silver sphere were obtained using the Mie theory [35]. The calculated spectra for the 40-nm silver sphere shows two resonance peaks: a main dipole resonance peak at 410 nm and a weaker quadrapolar resonance at 370 nm as a shoulder. The dipole resonance arises from one side of the sphere surface being positively charged, whereas the opposite side is negatively charged, giving the particle itself a dipole moment that reverses the sign at the same frequency as the incident light [36]. In Figure 2, it also presents a typical transmission electron microscopy image of the silver nanoparticles. It can be seen directly that the size of the nanoparticles is around tens of nanometers. Figure 2b shows the particle size distribution of 500 arbitrarily measured nanoparticles. The average particle size is around 70 nm. The larger particles shift the resonant wavelength to red [37]. Our results coincide well with the theoretical results.

**Figure 3:**
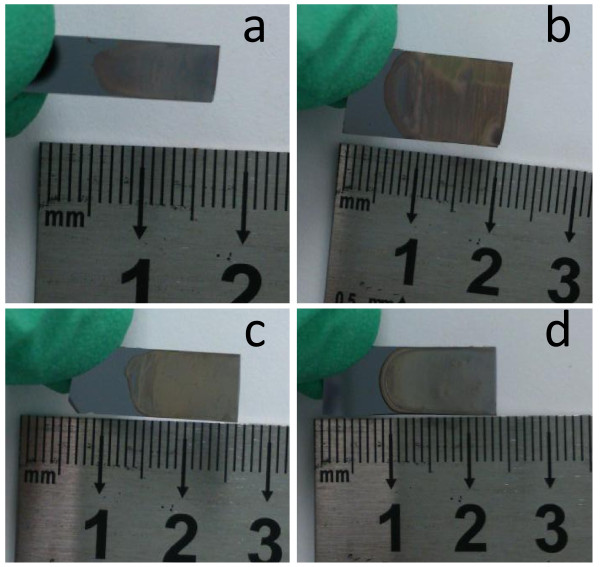
**Photos of silver nanoparticle film.** Prepared with different concentrations of silver nanoparticle solution: **(a)** 1 mM, **(b)** 10 mM, **(c)** 50 mM, and **(d)** 0.1 M.

Figure 3 shows the photos of silver nanoparticle film prepared with different concentrations of silver nanoparticle solution. It can be seen from Figure 3a that, at the concentration of 1 mM, only a circle pattern is formed on the edge of the solution. Because of the coffee ring effect, only a dense, ring-like deposit exists along the perimeter [23]. When the concentration is up to 10 mM, a grid-like film was formed on the surface of the wafer, as shown in Figure 3b. Continuing to increase the solution concentration in Figure 3c,d, a uniform thin film formed when the concentrations are 50 mM and 0.1 M. Except the part near the coffee ring which is not smooth, the rest of the film is smooth. The color of the film is silver-gray.

**Figure 4:**
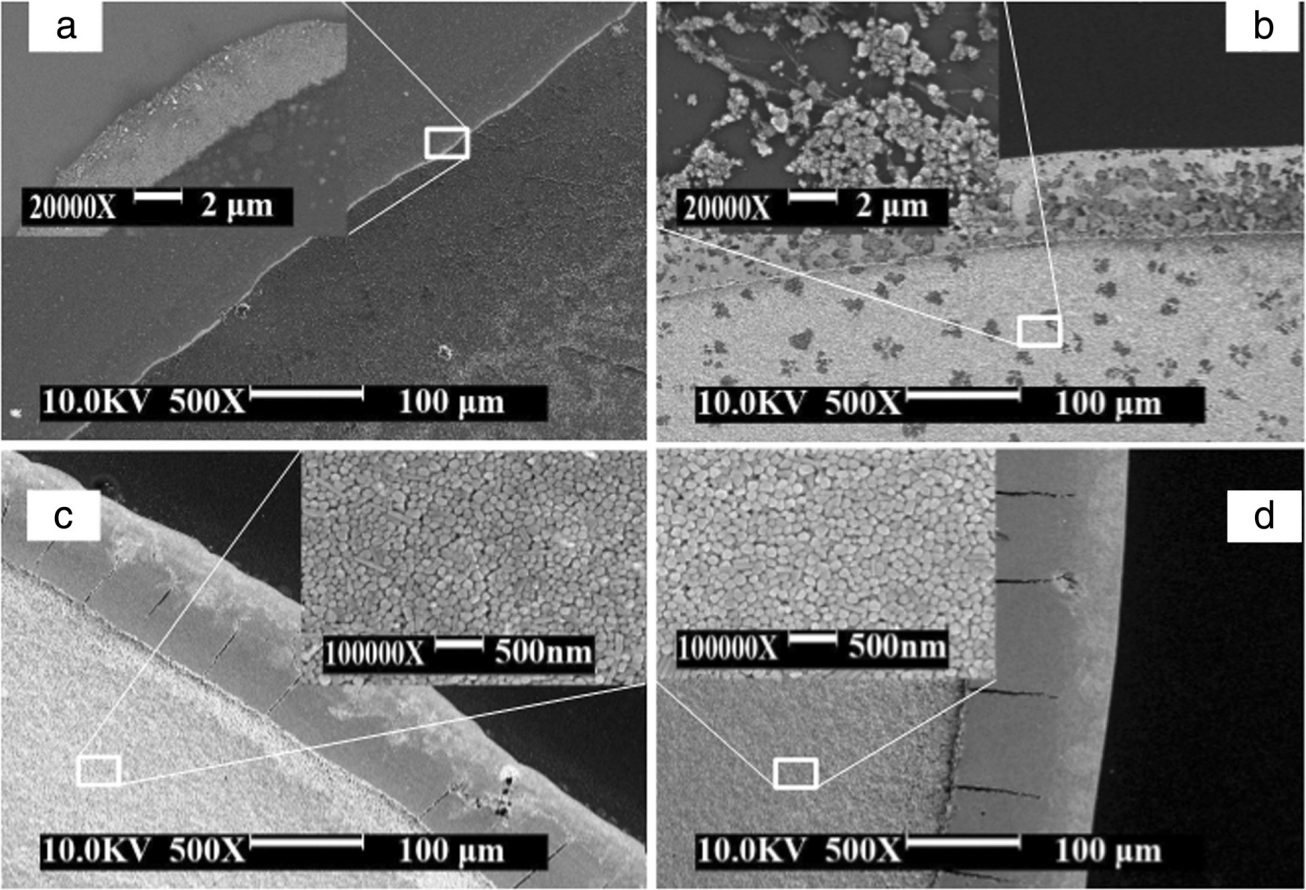
**Scanning electron microscope images of silver nanoparticle film.** Prepared with different concentrations of silver nanoparticle solution: **(a)** 1 mM, **(b)** 10 mM, **(c)** 50 mM, and **(d)** 0.1 M. The inset shows high-magnification SEM image of the film.

The scanning electron microscope images of silver nanoparticle films prepared with different concentrations of silver nanoparticle solution are displayed in Figure 4. From the scanning electron microscope images, one can see the morphology of the film obtained with coffee ring effect. It is obvious that there is only a circle pattern on the edge of the solution at the concentration of 1 mM from Figure 4a. A few silver nanoparticles were present inside the coffee ring. The width of the coffee ring is about 4 μm. When the concentration increases up to 10 mM, there is a coffee ring on the edge of the solution. Meanwhile, inside the coffee ring, there is a layer of silver thin film formed on the substrate. The local features can be seen from the inset of Figure 4b. The film is not uniform. These phenomena also appear in Figure 4c,d. However, it is notable that from the insets of Figure 4c,d, the film formed inside the coffee ring becomes smooth. Silver nanoparticles are uniformly distributed on the surface of the silicon substrate.

**Figure 5:**
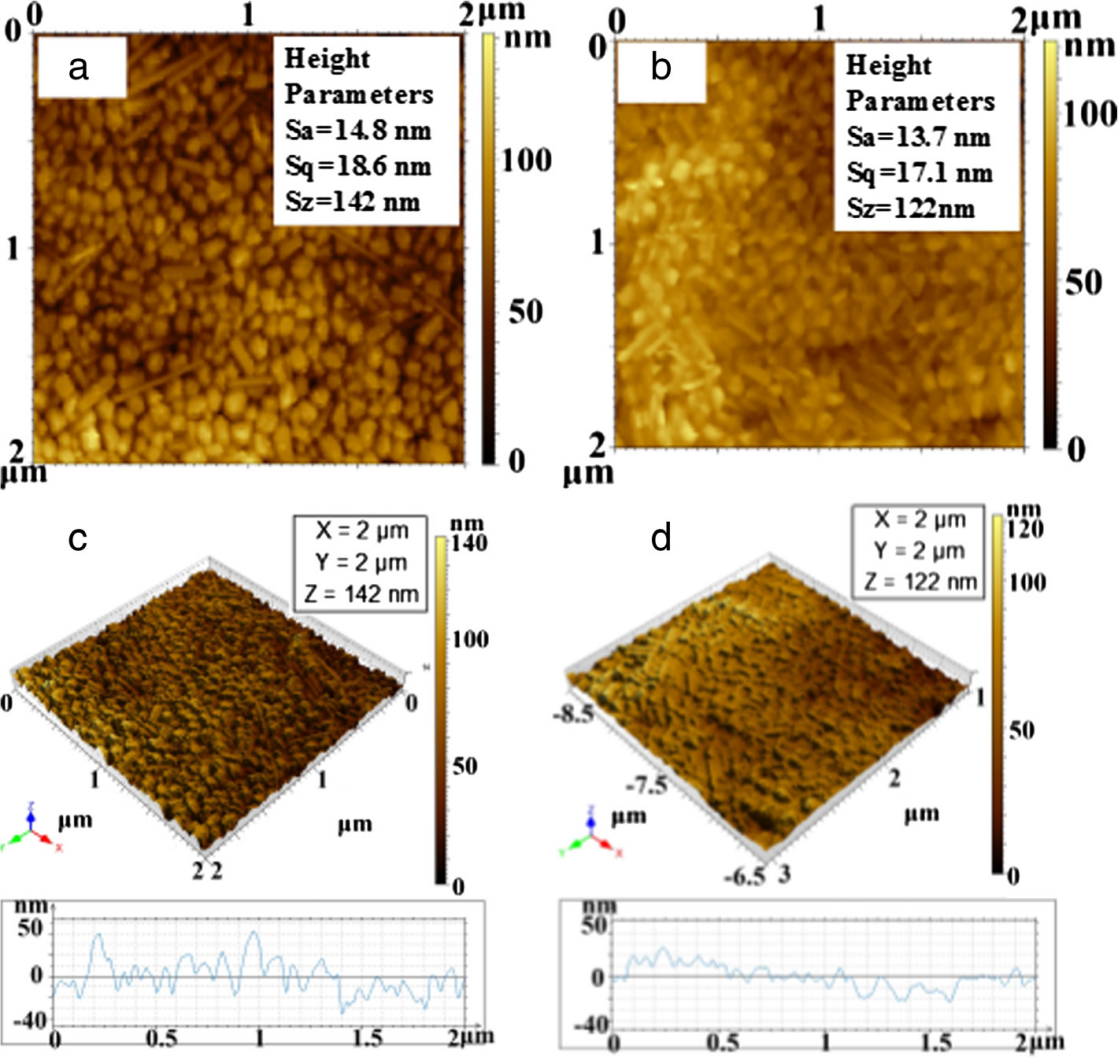
**Atomic force microscope images of silver nanoparticle film.** Prepared with the concentrations of silver nanoparticle solution of 50 mM **(a, c)** and 0.1 M **(b, d)**.

**Figure 6:**
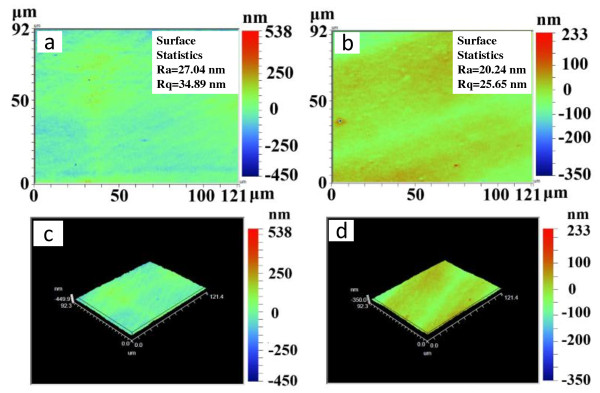
**Two-and three-dimensional surface profiles of the thin films.** Prepared with the solution of 50 mM **(a, c)** and 0.1 M **(b, d)**.

Figures 5 and 6 show the two- and three-dimensional surface profiles of the thin films using either a Veeco surface profiler or AFM. A Veeco surface profiler was used to detect the surface morphology at a larger area. Figure 5 shows the morphology features of the thin film at an area of 4 μm^2^. The surface roughness of arithmetical mean height (Sa) of the film prepared using the solution of the concentration from 50 mM to 0.1 M decreases from 13.7 to 14.8 nm. The root mean square heights (Sq) of the films are 17.1 and 18.6 nm, respectively. Quantitative characterization of the surface characteristics shows that the average roughness (Ra) of the film changes from 20.24 to 27.04 nm prepared using the solution of the concentration from 50 mM to 0.1 M. The root-mean-squared roughness (Rq) of the film shifts from 25.65 to 34.89 nm. The results obtained from the two methods are close. Quantitative characterization of the film by the two methods demonstrates that the film is very smooth.

Large-scale self-assembled silver nanoparticle films formed on the substrate are based on the modified coffee ring effect. When a drop of liquid dries on a solid surface, its suspended particulate matter is deposited in a ring-like fashion, forming a coffee ring [23,27]. In the present study, the conditions for forming the coffee ring were modified. At the concentration of silver nanoparticle solution ranging from 50 mM to 0.1 M with an incident substrate, smooth silver nanoparticle films can be obtained. The evaporating solution features an air-water interface shaped like a spherical cap. At the perimeter, the deposition of particles will pin the contact line, and thus, the radius of the liquid surface cannot shrink [23]. To realize this during evaporation, liquid must flow outwards. In practical, the liquid surface certainly decreases with the reduction of the solution. This results in the contact line moving inward. During the contact line movement, the capillary flow outward from the center of the solution brings suspended silver nanoparticles to the edge as evaporation proceeds [27]. Then, the self-assembled silver nanoparticles are deposited on the solid-liquid contact line. With the solid-liquid contact line moving inward, the silver nanoparticle film will be formed. Optimizedly, the decreasing speed of the liquid surface is synchronous with the forming velocity of solid films on the edge. At a low concentration of solution, almost all of the nanoparticles were deposited on the outer ring, causing no film generated inside, as shown in Figure 4a. Increasing the concentration up to 10 mM, scattering particles are deposited inside the ring. When the concentration is high enough, such as 50 mM or 0.1 M in our experiment, the silver nanoparticles promptly will fill the solid-liquid contact line and thereby form a smooth film.

**Figure 7:**
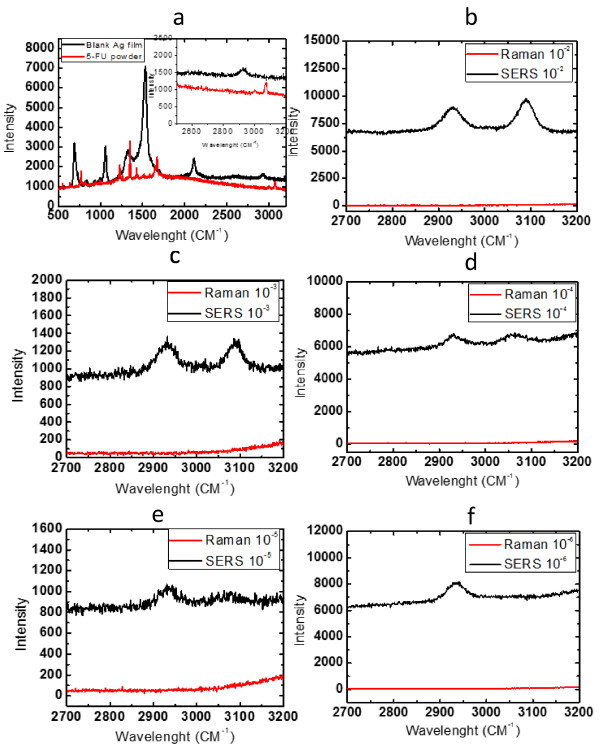
**Raman spectroscopy and surface-enhanced Raman spectroscopy.** 5-Fluorouracil solution and blank Ag film **(a)** (the inset shows the detail near 3,100 cm^−1^ with enlarged scale) and different concentrations **(b)** 1 × 10^−2^, **(c)** 1 × 10^−3^, **(d)** 1 × 10^−4^, **(e)** 1 × 10^−5^, and **(f)** 1 × 10^−6^. In **(b to f)**, the solid curve is the Raman spectroscopy of 5-fluorouracil solution on silver nanoparticle film, and the dash curve is the Raman spectroscopy of 5-fluorouracil solution on silica substrate.

The film prepared by this method was used as a Raman substrate. Figure 7 shows the Raman spectroscopy of 5-fluorouracil powder, silver nanoparticle film, and 5-fluorouracil solutions with different concentrations. The solid curve in Figure 7a is the Raman spectroscopy of blank silver nanoparticle films, and the dash curve is the Raman spectroscopy of 5-fluorouracil powder on silica substrate. Because 5-fluorouracil structure is a six-membered ring [38], the six-membered ring stretching vibrations are found in the region 3,125 to 2,925 cm^−1^[39]. In our experiment, a peak of 5-fluorouracil powder appears in 3,100 cm^−1^, while no peak appears at the same position of blank silver nanoparticle film. Thus, this peak is chosen as a characteristic peak of 5-fluorouracil. Figure 7b,c,d,e,f displays the Raman spectra of 5-fluorouracil solution with different concentrations. It can be seen from Figure 7b that, even at the concentration of 5-fluorouracil solution 1 × 10^−2^ M, there is no Raman signal of the solution dropped on silica substrate. However, there is a strong Raman peak of the solution dropped on silver nanoparticle film. When the concentration is down to 1 × 10^−5^ M, one can still obtain a Raman signal with silver nanoparticle films as substrates. When the concentration reaches to 1 × 10^−6^ M, all Raman peaks disappear with both kinds of substrates. It is clear that the silver nanoparticle film exhibits a good surface-enhanced Raman scattering effect. Farquharson et al. [38] researched the ability of SERS to measure the 5-fluorouracil in the saliva using silver-doped sol-gels which confirmed that the 5-fluorouracil samples of 2 μg mL^−1^ (1.5 × 10^−2^ M) were easily measured. Sardo et al. [40] obtained the SERS spectra of 5-fluorouracil recorded on silver sol and electrode of 10^−3^ M solution. In our experiment, the Raman signal can be detected in the solution with concentrations as low as 1 × 10^−5^ M. The apparent enhancement factor can be experimentally measured with direct comparison using the following relation: EF = (RS^ENH^/RS^REF^) × (*C*^REF^/*C*^ENH^), where RS^ENH^ and RS^REF^ are the measured Raman intensities and *C*^REF^ and *C*^ENH^ are the solution's concentrations for normal and enhanced samples [41]. The 5-fluorouracil Raman scattering signals on the surface of the silver nanoparticle film exhibit a cross-sectional enhancement factor up to 1.08 × 10^4^. In our experiment, the concentration of solution 1 × 10^−1^ M was not obtained because of the low solubility. Thus, the enhancement factor may be higher than 1.08 × 10^4^. From the results we obtained, the film can successfully be used in the detection of the low concentration medicine. With the further optimization, this technique may be utilized in biochemical and trace analytical applications.

## Conclusions

An innovative concept of preparing silver nanoparticle films based on the coffee ring effect using the surface-enhanced Raman spectroscopy for the detection of the low-concentration medicine is demonstrated. Silver nanoparticles with the average size about 70 nm were prepared by reduction of silver nitride. In our experiment, the coffee ring effect was controlled and used for preparing silver nanoparticle films. The silver nanoparticles were spontaneously formed on the surface of the silicon substrate at the temperatures about 50°C based on the coffee ring effect. The quantitative characterization of the surface characteristics shows that the average roughness of the film is from 20.24 to 27.04 nm prepared using the solution of the concentration from 50 mM to 0.1 M. It is evident that the silver nanoparticle film exhibits the remarkable surface-enhanced Raman scattering effect. Raman signal can be detected in the 5-fluorouracil solution with concentrations as low as 1 × 10^−5^ M, and the enhancement factor achieved by the silver nanoparticle film can be higher than 1.08 × 10^4^. Our experimental results show great promise in the production of large-scale silver nanoparticle films for the surface-enhanced Raman scattering.

## Competing interests

The authors declare that they have no competing interests.

## Authors’ contributions

WZ and AH conceived of the study and drafted the manuscript. SB helped with the preparation of silver nanoparticles. YM helped with the Veeco characterization. QS helped with the SEM characterization. All the other works were carried out by WZ. All authors read and approved the final manuscript.
